# Onasemnogene Abeparvovec Administration via Peripherally Inserted Central Catheter: A Case Report

**DOI:** 10.3390/children11050590

**Published:** 2024-05-13

**Authors:** Inmaculada Pitarch Castellano, Eduardo López Briz, Eugenia Ibáñez Albert, Cristina Aguado Codina, Teresa Sevilla, José L. Poveda Andrés

**Affiliations:** 1 Department of Pediatrics, Hospital Universitario y Politécnico la Fe, 46026 Valencia, Spain; 2 Department of Pharmacy, Hospital Universitario y Politécnico la Fe, 46026 Valencia, Spain; lopez_edubri@gva.es; 3 Department of Physical Medicine & Rehabilitation, Hospital Universitario y Politécnico la Fe, 46026 Valencia, Spain; ibanyez_eug@gva.es; 4 Department of Clinical Analysis, Hospital Universitario y Politécnico la Fe, 46026 Valencia, Spain; aguado_cri@gva.es; 5 Department of Neurology, Hospital Universitario y Politécnico la Fe, 46026 Valencia, Spain; sevilla_ter@gva.es; 6 Management Department, Hospital Universitario y Politécnico la Fe, 46026 Valencia, Spain; poveda_josand@gva.es

**Keywords:** onasemnogene abeparvovec, peripherally inserted central catheter, adeno-associated gene therapy, spinal muscular atrophy, adverse events

## Abstract

Onasemnogene abeparvovec (OA) is the approved intravenous gene therapy for the treatment of spinal muscular atrophy (SMA). A functional copy of the human *SMN1* gene was inserted into the target motor neuron cells via a viral vector, AAV9. In clinical trials, OA was infused through a peripheral venous catheter, and no data are available on central catheter use. Recently, we had a case where OA was administered directly into the right atrium via a peripherally inserted central catheter (PICC) instead of a peripheral line, as recommended. The patient was a female child aged 4 months, diagnosed as SMA type I. For practical reasons, a dose of OA according to the weight of the patient (1.1 × 10^14^ vectorial genomes/kg) was administered via PICC in 1 h, as the product information recommends. The drug was well tolerated, with no hypersensitivity reactions or initial elevation of transaminases or other adverse effects. To our knowledge, this is the first case reported where OA was administered via a central line. This type of administration is not contraindicated, but it is not specifically contemplated or recommended. It is unknown whether central line administration could have any implications for transduction efficiency and immunogenicity. Future studies should clarify these aspects, as each gene therapy has a specific optimal dose recorded that depends on the site and route of administration of the drug, the AAV variant and the transgene.

## 1. Introduction

Spinal muscular atrophy (SMA) is a rare genetic disease occurring in one out of every 10,000 live births. Type I SMA shows up before 6 months of age and is the most severe form of disease, being rapidly fatal if untreated. In May 2019, the Food and Drug Administration (FDA) (in May 2020, the European Medicines Agency, EMA) approved onasemnogene abeparvovec (OA) for the treatment of type I SMA, patients with 5q SMA with a bi-allelic mutation in the SMN1 gene and up to 3 copies of the *SMN2* gene [1,2]. Treatments involve a single intravenous administration. A functional copy of the human *SMN1* gene was introduced into the target motor neuron cells via a viral vector, AAV9, resulting in increased expression of the SMN protein gene in all cells, including alpha motor neurons. OA uses the AAV9 viral vector, since it can cross the blood–brain barrier; the transgene will not integrate into the nucleus of the cells, but will remain as an extrachromosomal episome, using the cell’s transcription to produce the functional SMN protein [3].

The manufacturer recommends a dose of 1.1 × 10^14^ vector genomes per kilogram (vg/kg) of body weight. In clinical trials, OA was infused through a peripheral venous catheter, and no data are available on central catheter use [4]. OA is currently under research to explore the intrathecal route, with the aim to reduce the total amount of dose and reduce the adverse events related to the systemic administration of the therapy [5].

## 2. Case Report

A patient treated with OA, administered via a peripherally inserted central catheter (PICC) instead of a peripheral line, as recommended, is presented.

This is a case of a female child born with normal prenatal and perinatal periods. She had two episodes of bronchiolitis at 2 and 4 months. At the last admission, SMA was suspected based on delayed head control, proximal muscle weakness, and lingual fasciculations. An *SMN1* deletion was found, along with two copies of the *SMN2* gene, resulting in a diagnosis of SMA type 1.

The parents were informed by the multidisciplinary team in pediatric neuromuscular diseases of the available options, the benefit/risk ratio of each drug, as well as the conditions for treatment follow-up, following the Delphi consensus on recommendations for the treatment of spinal muscular atrophy in Spain (RET-AME consensus) [6]. The family choose OA gene therapy from the available options (nusinersen and risdiplam). The patient’s anti-AAV9 antibody test results were negative. Informed consent for treatment was obtained, following the pharmacoclinical protocol for treatment with OA (Zolgensma^®^) in patients with spinal muscular atrophy in the national health system [7]. Motor function was assessed with the Children’s Hospital of Philadelphia Infant Test of Neuromuscular Disorders (“CHOP INTEND”) of twenty-one points, normal cardiac function, and compound muscle action potential (CMAP) of the ulnar nerve amplitudes of 1.2 mV. Her weight at the time of infusion was 4.8 kg.

As a result of the previous viral infection that caused bronchiolitis, the infant had elevated levels of aspartate aminotransferase (AST) 91 U/L (normal value 5–34), 996,000 platelets per microliter (normal range 150,000–500,000 platelets per microliter) and Troponin T Ultrasensitive 19.40 ng/L (normal value 0.00–14.00), with a normal level of alanine aminotransferase (ALT) 23 U/L (normal value 0–55), the day before the planned OA administration.

For practical reasons, a PICC was inserted on the day of OA administration, and a dose of OA according to the weight of the patient (1.1 × 10^14^ vectorial genomes/kg) was administered via this PICC in 1 h, as product information recommends. The drug was well tolerated, with no hypersensitivity reactions or initial elevation of transaminases.

On day +1, liver enzyme values had dropped, with AST 37 U/L and ALT 18 U/L, just as platelets had dropped to 795,000/μL, and only Troponin T Ultrasensitive increased to 38.00 ng/L. On day +5, we were able to verify the rise of the liver enzymes AST 217 U/L and ALT 105 U/L, the decrease in the Troponin T Ultrasensitive to 16.80 ng/L and the normalization of platelets at 198,000/μL. Liver enzymes progressively decreased to normalization on day +14.

No monitored adverse effects (vomiting, fever, signs of cold, food refusal, altered bowel movements or skin changes) or other adverse effects were recorded during the two-month follow-up period. The patient’s motor skills were assessed with the CHOP INTEND scale after the administration of OA, showing an increase of 3 baseline points at 1 month and 7 baseline points at 2 months (in clinical trials with OA, an increase of 4 points in the year following the infusion is considered a positive response) [8,9].

## 3. Discussion

Intravenous gene therapy with AAV vectors is being assessed in diseases other than SMA, but a better understanding of the immune response to prevent liver injury is still needed [10]. Adverse effects that should be controlled with OA include acute liver injury, elevated troponin I, transient thrombocytopenia, and thrombotic microangiopathy. The most common serious effects reported are liver effects [11].

Hepatotoxicity associated with gene therapy has been observed with AAV9 vectors administered intravenously and intrathecally. In non-human primates, these transient elevations of markers of acute liver injury occur within 3–4 days after intravenous administration or about two weeks after intrathecal administration by complement activation and are due to transduction of the transgene. This acute liver injury has been attributed to high hepatocellular vector load, macrophage activation, and innate pathway responses to interferon virus type 1 [12].

The most striking thing about the case was that the transaminase values decreased on the first day after the PICC infusion, since in clinical practice what we have observed after peripheral infusion is always an increase, whether significant or not. This first-day increase is presumed to be due to hepatocellular uptake of AVV9, as the liver is among the most transduced tissues [13]. And although the mechanism of OA-related liver damage is unknown, it is assumed that the greater the hepatocellular uptake of AVV9, the greater the hepatic involvement.

A variety of short-, moderate, and long-term vascular access devices are available (i.e., midline catheters, central venous catheters, PICCs, peripheral intravenous [IV] catheters, ports, etc.). PICC is a type of central catheter, commonly used in adults and children; PICC lines provide options that routine peripheral intravenous access or other devices do not provide and without the risks associated with direct puncture of central vessels. A PICC is inserted into a peripheral vein (basilic, cephalic, or brachial veins), and then guided toward the heart, until its end is in the lower third of the superior vena cava towards the cavo-atrial junction [14]. Although a PICC is inserted in a peripheral vein, the tip reaches a central vessel. While midline catheters and peripheral intravenous cannulas are restricted to infusions of drugs and fluids with a pH close to 7 (5–9) and osmolarity of up to 500–600 mOsm/L, PICCs can be used as central lines, regardless of pH and osmolarity of the solution administered, and can avoid endothelial damage induced by hyperosmolar drugs or by those with extreme pH.

Intravenous administration of gene therapies is the most common route of administration, due to its efficacy in clinical trials, which is the highest of all routes (80% success rate). However, intravenous administration also requires higher doses than other routes (up to 4.4 × 10^13^ vg/kg per patient) and involves greater risks: the higher proportion of treatment-emergent serious adverse events (TESAEs) correspond to clinical trials where gene therapy was administered intravenously, since administration through the bloodstream results in greater distribution to off-target tissues and organs [15].

To our knowledge, this is the first reported case in which OA was administered through a central line. This type of administration is not contraindicated in the manufacturer’s product information but is not specifically contemplated or recommended in the manufacturer’s product information. Based on clinical perception of reduced adverse effects after central infusion, we conducted a literature search, but could not identify any relevant reports in which a central line had been used to deliver gene therapy. In clinical trials of intravenous administration, OA was infused through a peripheral venous catheter.

In our patient, two peripheral accesses were first channeled (one in the hand and the other at the cephalic level), but neither of them gave us the total confidence to infuse a gene therapy without interruptions or setbacks. So, it was decided a few hours before the infusion to channel better access. Our hospital has a qualified team of doctors and nurses for the placement of the PICC line using fluoroscopic guidance with great clinical experience and with a very low rate of vascular or infectious complications, which gives us great peace of mind and confidence in its use.

PICC lines are indicated in situations where long-term vascular access is needed or when a safer and more efficient route of administration is required [16]. Central lines are ideal for the administration of medications that may be irritating to the peripheral veins or that must be administered at a controlled and constant rate. In our case, it was only required for an infusion of 1 h, but it was decided to place it because the patient had poor peripheral access. During the previous hospitalization for bronchiolitis, multiple peripheral accesses had been channeled with great difficulty and with few guarantees of permanence. In addition, we know that patients with severe forms of SMA (SMA type 1) have frequent vascular [17] or cardiac [18] defects, so a cardiological examination was performed prior to the implantation of the PICC, which was normal, and no contraindications for its placement were detected [16].

Assessment of liver function is particularly important given that patients with SMA may be at increased risk of liver dysfunction. Our patient had a baseline ALT elevation greater than twice the upper limit of the normal value. Although we cannot draw general conclusions from a simple case, we wonder if the only change we have made with respect to the other cases has been influential, which has been to infuse OA into the right atrium instead of infusing peripherally into the slow-return venous circulation. The administration of OA directly in the right atrium, with the affinity of the AAV9 viral vector at the cardiac level, was a challenge; we think that the mild cardiac lesion detected may be due to the infant’s own physiology, with normal heart rates at this age between 90–180 bpm. The patient pumped at 175 bpm throughout the infusion. Thus, OA rapidly crosses the blood–brain barrier into motor neurons, and passage through the liver is ultimately relegated.

We know that the optimal dose is specific to each gene therapy that has been registered and depends on many factors, including the AAV variant, the transgene itself, but interestingly, also the route and site of administration of the drug [19]. In the case of OA, the indication is intravenous, but there are no data on its central administration. After the experience of this case, administered centrally without clinical side effects, with few laboratory alterations and with a good motor response, we wonder if the uptake of OA by the motor neurons of the anterior horn of the medulla can be greater when administered centrally or if the hepatic involvement may be less.

Therefore, more controlled studies, focusing on transduction efficiency and immunogenicity, should be conducted in animal models to know the optimal doses for different AAV variants. These data could be used to develop guidelines and future assays focused on the knowledge of the best dosing regimens for selected AAVs, to maximize the clinical efficacy and thus the clinical success of AAV gene therapy. More work is needed to better understand the immune response in the context of intravenous gene therapy with AAV vectors and to learn how to optimally prevent liver injury.

## Data Availability

The data that support the findings of this study are available on request from the corresponding author. The data are not publicly available due to privacy or ethical restrictions.
